# X-ray diffraction studies on merohedrally twinned Δ1–62NtNBCe1-A crystals of the sodium/bicarbonate cotransporter

**DOI:** 10.1107/S1744309113016710

**Published:** 2013-06-28

**Authors:** Harindarpal S. Gill, Lauren Dutcher, Walter F. Boron, Samir Patel, Lisa M. Guay-Woodford

**Affiliations:** a Department of Medicine, The George Washington University, Washington, DC 20052, USA; b Division of Renal Diseases and Hypertension, The George Washington Medical Faculty Associates, Washington, DC 20037, USA; c Department of Physiology and Biophysics, Case Western Reserve University School of Medicine, Cleveland, OH 44106, USA; d Hospital of University of Pennsylvania, Philadelphia, PA 19104, USA; e Center for Translational Science, Children’s National Medical Center, Washington, DC 20010, USA

**Keywords:** NBCe1, bicarbonate transport

## Abstract

NBCe1-A membrane-embedded macromolecules that cotransport sodium and bicarbonate ions across the bilayer serve to maintain acid–base homeostasis throughout the body. Defects result in a number of renal and eye disorders, including type-II renal tubular acidosis and cataracts. Here, crystals of a human truncated mutant of the cytoplasmic N-terminal domain of NBCe1 (Δ1–62NtNBCe1-A) are reported that diffract X-rays to 2.4 Å resolution. The crystal symmetry of Δ1–62NtNBCe1-A is of space group *P*3_1_ with pseudo-*P*3_1_21 symmetry and it has a hemihedral twin fraction of 33.0%. The crystals may provide insight into the pathogenic processes observed in a subset of patients with truncating and point mutations in the gene encoding NBCe1.

## Abstract 2

A truncated mutant missing the first 62 residues of the N-terminal, cytoplasmic domain of the sodium-bicarbonate NBCe1-A cotransporter crystallizes in space group *P*3_1_ with pseudo-*P*3_1_21 symmetry and a hemihedral twin fraction of 33.0%. Twinned fractions and twin-pair statistics over binned resolutions confirm that the calculated twin fraction is associated with hemihedral twinning and not to non-crystallographic symmetry.

## 1. Introduction

Bicarbonate (HCO_3_^−^) transport in the kidney is critical for acid–base balance in the body. Every day, normal kidneys filter and reabsorb approximately 4 mol (250 g) of HCO_3_^−^. The proximal tubule is the site of the kidney that reabsorbs 80% of the filtered HCO_3_^−^ load. Impaired bicarbonate reabsorption in the proximal tubule, diagnosed in patients as proximal renal tubular acidosis (pRTA or type-II RTA), is characterized by hyperchloremic metabolic acidosis with variable hypokalemia (Haque *et al.*, 2012 ▶). Na^+^-coupled HCO_3_^−^ transporters (NCBTs) are integral membrane proteins that are responsible for handling (either reabsorbing or secreting) Na^+^ and HCO_3_^−^ ions in tissues throughout the body. NBCe1-A is the major electrogenic NCBT transporter found at the basolateral membrane of the proximal tubule that mediates the crucial step in the transepithelial movement of HCO_3_^−^ ions, thus maintaining blood pH. Studies in patients with isolated type-II RTA have led to the identification of 11 natural occurring mutations in NBCe1-A (Igarashi *et al.*, 1999 ▶), two of which are Q29X and R298S in the cytoplasmic N-terminal domain (NtNBCe1-A). Most patients with NtNBCe1-A mutations have type-II RTA and in addition exhibit systemic manifestations including mental retardation, growth retardation and myriad ocular defects, *e.g.* band keratopathy, glaucoma and cataracts (Suzuki *et al.*, 2012 ▶). These findings underlie the need to understand how NtNBCe1 may function in other tissues such as the corneal epithelium, lens epithelium and ocular ciliary epithelium, where it has recently been identified (Suzuki *et al.*, 2012 ▶), and how mutations cause disease.

Protein crystals of the *P*3 subset of trigonal space groups are commonly associated with merohedral twinning (Yeates & Tsai, 2011 ▶; Yeates, 1997 ▶; Sawaya, 2007 ▶; Berthold *et al.*, 2006 ▶). They are the most complicated case of twinning because not one but three sets of twin operators are possible, giving rise to apparent crystal point groups 321, 312 or 6 (Yeates & Tsai, 2011 ▶; Sawaya, 2007 ▶). Many programs to identify twinning have difficulty in distinguishing noncrystallographic symmetry (NCS) from merohedral twinning. Thus, twin fractions tend to be overestimated (Yeates & Tsai, 2011 ▶). In order to identify and calculate a conservative twin fraction, a partial twinning test for two-domain merohedral twinning (referred to as hemihedral twinning) can be implemented that evaluates the variable *H* (*i.e.* the difference between intensities of twin pairs divided by their sum) as a function of resolution (Yeates & Tsai, 2011 ▶) and this service is available at the NIH–UCLA twin server (Yeates & Fam, 1999 ▶). To complicate matters even more, NCS operators often also tend to coincide with crystallographic symmetry operators, resulting in space-group ambiguity (Sawaya, 2007 ▶). In the previous study of full-length NtNBCe1-A crystals that diffracted X-rays to 3.0 Å resolution (Gill & Boron, 2006*a* ▶), the self-rotation function at the κ = 180° section demonstrated dyads in the ab* plane spaced approximately every 60° along ψ starting at (30, 0°), giving rise to space-group ambiguity between *P*3_1_21 and *P*3_1_ with pseudo-*P*3_1_21 symmetry. Assuming that the dyads were not the expected crystallographic peaks of the *P*3_1_21 space group, they confirmed the presence of a twofold NCS element expected for a dimeric NtNBCe1-A as estimated in Gill & Boron (2006*b* ▶) and accurately measured in Gill (2012 ▶). In this study, we characterize crystals of Δ1–62NtNBCe1-A that diffract X-rays to 2.4 Å resolution, despite the detection of partial merohedral twinning, anisotropy and persistent space-group ambiguity.

## 2. Materials and methods

### 2.1. Expression, purification and crystallizations

**Figure 1:**
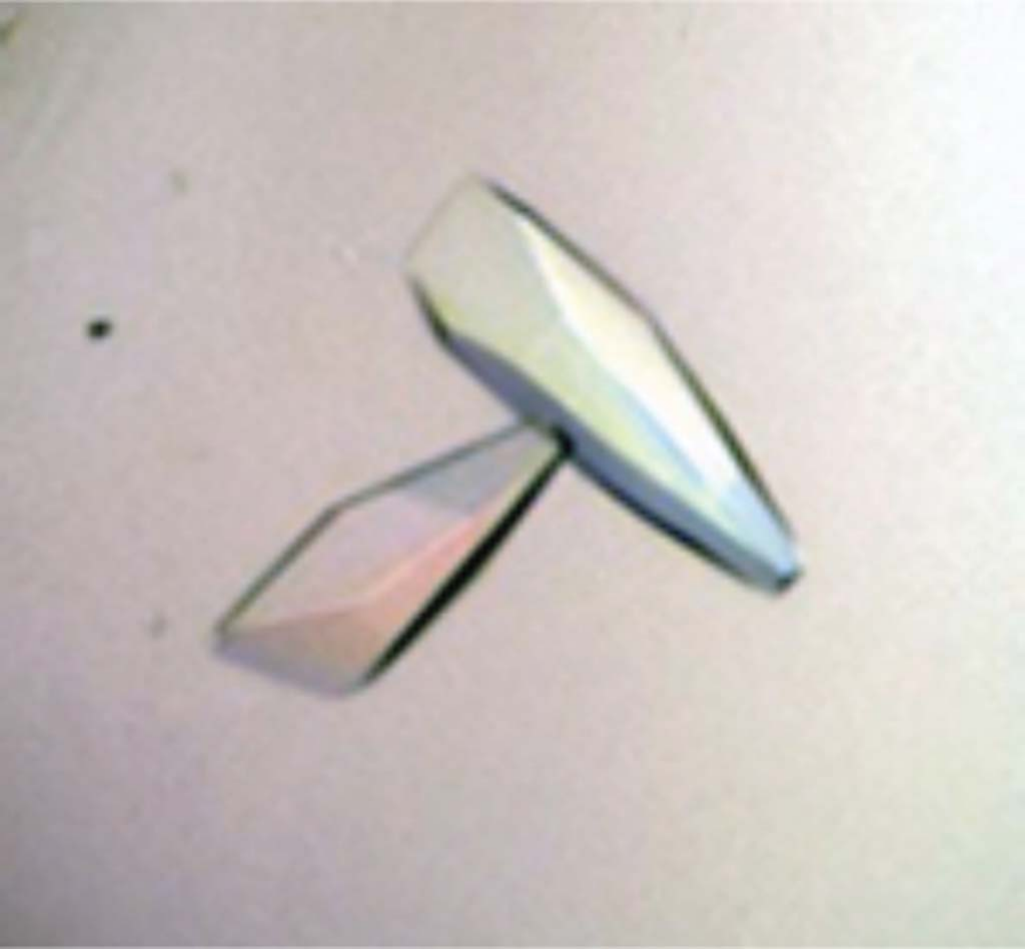
Crystal of truncated NtNBCe1. The crystals formed into a wedge shape with a variety of sizes. Those shown are colored by a polarization filter, have dimensions of 0.01 × 0.01 × 0.03 mm, grew from a hanging-drop screen using a nano-drop liquid handler and are morphologically similar to those in this study.

The expression and purification of full-length Nt (residues 1–362) have previously been described in Gill & Boron (2006*b* ▶) and subsequently refined in Gill (2012 ▶). Crystal conditions have previously been described in Gill & Boron (2006*a* ▶). To generate better X-ray diffracting crystals, a truncated mutant lacking the first 62 residues (Δ1–62NtNBCe1-A) was recombinantly expressed and purified using similar procedures and conditions as the full-length Nt. Δ1–62NtNBCe1-A was crystallized by hanging-drop vapor-diffusion methods (McPherson *et al.*, 1995 ▶). Each drop had a total volume of 4 µl and consisted of equal parts of well solution and a 30 mg ml^−1^ NtNBCe1 stock solution. The volume of the well was 0.5 ml. The mother solution in the well consisted of 35%(*v*/*v*) saturated ammonium sulfate solution in 150 m*M* sodium citrate pH 6.5, similar to that used for the full-length Nt (Gill & Boron, 2006*a* ▶), yielding wedge-shaped crystals as shown in Fig. 1 ▶.

### 2.2. X-ray data collection and processing

The procedures for data collection from Nt crystals have been described previously (Gill & Boron, 2006*a* ▶). For Δ1–62NtNBCe1-A crystals, data were collected on the X29 beamline using a Quantum 3 × 3 detector (Area Systems Detector Corporation, San Diego, California, USA) at the National Synchrotron Light Source at Brookhaven National Laboratory, Long Island, New York, USA. At the synchrotron, a Δ1–62NtNBCe1-A crystal was flash-cooled by swiping the crystal in bicycle oil (Tri-Flow superior lubricant with Teflon; Sherwin-Williams Consumer Group, Cleveland, Ohio, USA) and immediately placing the crystal in a cryostream. Data were collected initially to 2.6 Å resolution and then subsequently to 2.4 Å resolution after annealing the crystal a few times (*i.e.* thawing the flash-cooled crystal in mother liquor for ∼3 min and then again flash-cooling in the cryostream). The crystal was oscillated through 60° (assuming a *P*3 space group) and data collection then continued with an oscillation angle of 0.8°. Each frame was exposed for 3 s. The crystal-to-detector distance was set to 325 mm. Data were processed using the program *HKL*-2000 (Otwinowski *et al.*, 2003 ▶). Merohedral twinning was validated by calculating the variable *H* as a function of resolution using the NIH twinning server (http://nihserver.mbi.ucla.edu/Twinning; Yeates & Fam, 1999 ▶). Anisotropic corrections were applied to the Nt and the Δ1–62NtNBCe1-A data sets using the server at http://services.mbi.ucla.edu/anisoscale (Strong *et al.*, 2006 ▶). After anisotropic scaling, isotropic *B* factors of −27.24 Å^2^ for the previously collected Nt data set and −20.94 Å^2^ for the data set from Δ1–62NtNBCe1-A were applied to restore the magnitude of the high-resolution reflections as described in Strong *et al.* (2006 ▶).

## 3. Results and discussion

### 3.1. Crystal parameters

Data collected to 3.0 Å resolution from the full-length Nt crystal have been characterized in Gill & Boron (2006*a* ▶). These reported NtNBCe1-A molecules crystallized in a trigonal space group, yielding equal possibilities of space group *P*3_1_ or *P*3_1_21. For either space group, the crystal had unit-cell parameters *a* = *b* = 51.7, *c* = 200.6 Å, where the smaller dimensions roughly reflect the diameter of the dimer near neutral pH. Using a monomer of the N-terminal domain of family member AE1 (NtAE1; Zhang *et al.*, 2000 ▶) as a probe in molecular-replacement methods, the crystal-packing solution in either space group yielded an arm-swapped dimer in character with NtAE1. For the *P*3_1_ possibility, the entire dimer is contained in the asymmetric unit. For *P*3_1_21, the twofold symmetry of the dimer coincides with a crystallographic axis. The space group was to be sorted out by model refinement. However, this ultimately was not possible owing to a poor data-to-parameter ratio. Subsequently, the annealed Δ1–62NtNBCe1-A crystal diffracted X-rays to 2.4 Å resolution. Data reduction indicated the same apparent space-group ambiguities as the full-length Nt except that the unit-cell parameters were *a* = *b* = 54.0, *c* = 398.4 Å, approximately doubled along the *c* axis. In a *P*3 space group, a calculated Matthews coefficient (*V*_M_; Kantardjieff & Rupp, 2003 ▶; Matthews, 1968 ▶) of 2.39 Å^3^ Da^−1^ (monomer molecular weight 35 073 Da) indicates four monomer molecules in the asymmetric unit; in a *P*321 space group, the *V*_M_ indicates two monomer molecules in the asymmetric unit.

### 3.2. Resolution and self-rotation functions

Annealing the Δ1–62NtNBCe1-A crystal apparently improved the X-ray diffraction from 2.6 to 2.4 Å resolution. Data re-collection and reduction suggest that both versions have the same possible space groups within the trigonal set and with similar unit-cell parameters. The κ = 180° section of the self-rotation functions after annealing reveals that the highest set of dyads have a 5° offset from the crystallographic b* axis, indicating they arise from NCS. With two dimers calculated to be in the asymmetric unit from a necessary *P*3, *P*3_1_ or *P*3_2_ space group, the κ = 180° section suggests two overlapping twofold elements whose NCS axes (presumably from two biological dimers) are parallel to one another.

### 3.3. Merohedral twinning

**Figure 2:**
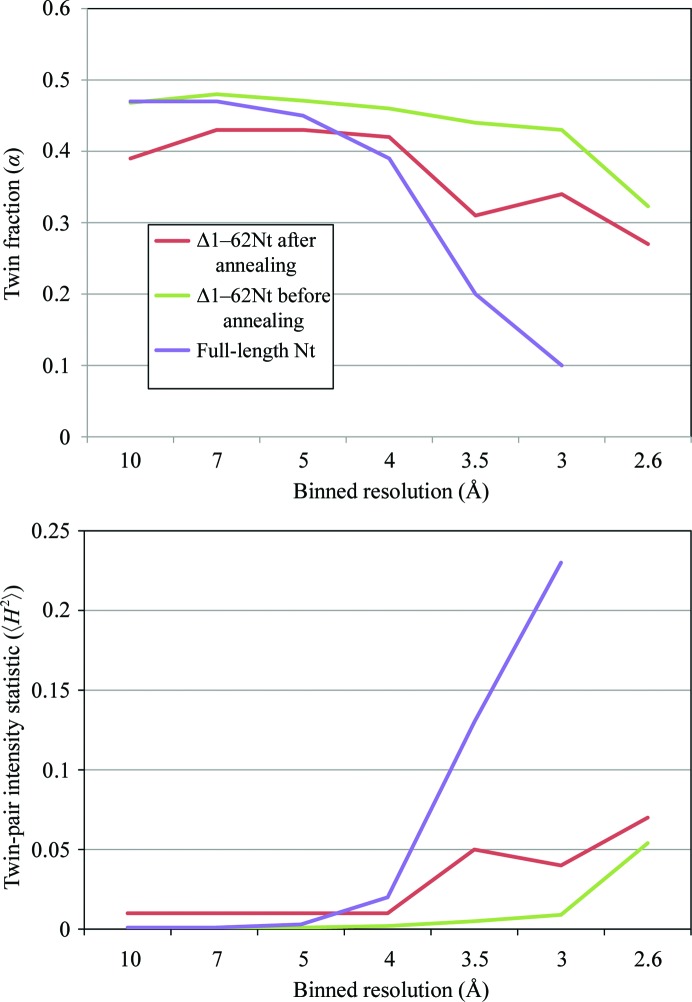
*H* *versus* resolution twinning test. The binned twinning-test calculations are shown for the Δ1–62NtNBCe1-A crystal and the full-length Nt crystal for twin operator 2 along *a*, *b*. As described in the text, the trends demonstrate that the calculated twin fractions are associated with hemihedral twinning and not NCS (green and red curves). In contrast, the binned twinning test for the full-length Nt crystal (purple curves) suggests little to no twinning despite an apparent twin-fraction calculation of 33.1% over the entire unbinned resolution range. Note that the Δ1-62NtNBCe1-A crystal before annealing has an apparent higher twin fraction of the crystal compared with that after annealing, either indicating a dramatic physical change in the lattice after annealing or a non-uniform crystal lattice.

The Δ1–62NtNBCe1-A crystal before annealing had an estimated hemihedral twin fraction of 43.6% with twin operator 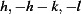 (or the symmetry-related operator 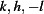) in reciprocal space. The crystal after annealing was calculated to have a hemihedral twinning fraction of 33.0%, again with twin operator 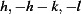. The values for α are calculated for the entire resolution range (34.1–2.41 Å) and are in agreement with the refined α calculated using *PHENIX* (Adams *et al*., 2010 ▶). The data-collection statistics are shown in Table 1 ▶. Twin fraction α and twin-pair intensity statistic *H* values over binned resolutions are illustrated in Fig. 2 ▶. Here, a conservative resolution limit of 2.6 Å is used in the calculations for the Δ1–62NtNBCe1-A crystal. As illustrated, partial twinning is observed as the estimates of α remain significantly above zero and 〈*H* ^2^〉 tends to be similar out to high resolution (green and red curves). In the case of NCS but no twinning, the similarity between twin pairs (to a degree that depends on α) tends to drop off strongly as a function of resolution, usually by 3 Å (T. O. Yeates, personal communication). This is exemplified by the full-length Nt crystal (Gill & Boron, 2006*a* ▶). The overall (44.8–3 Å) twinning fraction appears to be 33.1% in the data set comprising the first 120° of oscillations (increasing to 42.4% with 359° of oscillations). However, looking at the split shells out to higher resolution, the apparent twin fraction is significantly overestimated owing to NCS, which is deduced by the drop off in α and the sharp increase in 〈*H* ^2^〉 (purple curves).

### 3.4. Anisotropy

Annealing also reduced the anisotropy of the NtNBCe1-A and Δ1–62NtNBCe1-A crystals. Full-length NtNBCe1-A crystals initially did not diffract X-rays. After annealing, a crystal diffracted X-rays to 3.0 Å resolution. The collected data had strong anisotropy (Δ*B* of 40.54 Å^2^). The data from a Δ1–62NtNBCe1-A crystal before annealing also had strong anisotropy (Δ*B* of 38.82 Å^2^). The same Δ1–62NtNBCe1-A crystal after annealing shows that the anisotropy is mild (Δ*B* of 21.52 Å^2^). No truncations were necessary.

### 3.5. Molecular packing

Inspection of systematic absences did not help to reduce the space-group possibilities, presumably as a consequence of the twinning. However, molecular-replacement searches using *Phaser* (McCoy *et al.*, 2007 ▶) yielded the highest peaks in *P*3_1_ with a *Z*-score of 15. Searches in *P*3 and *P*3_2_ did not yield solutions. The *Z*-score of searches in all trigonal space groups are shown in Table 2 ▶. The packing solution for the full-length Nt and Δ1–62NtNBCe1-A crystals show a similar mechanism for dimerization, *i.e.* two interlocking domains at the C-terminal end. The two arm-swapped dimers have their twofold axes parallel to each other, both aligned with the crystallographic *a* axis.

### 3.6. Purification and other challenges with the Nt

The easiest way to overcome merohedrally twinned crystals is to find another space group whose point-group symmetry does not lend itself to this sort of twinning or to screen many crystals in the problematic crystal system in the hope of finding a crystal with a single domain. These possibilities did not lend themselves to Δ1–62NtNBCe1-A, and only a very limited number of crystals grew to sufficient size for either Nt or Δ1–62NtNBCe1-A. Growing reproducible Nt and Δ1–62NtNBCe1-A crystals has been challenging for a number of reasons. Isolated molecules often lead to peculiar discrete waves of precipitation (Gill & Boron, 2006*b* ▶). Precipitation can be overcome by keeping the protein in a taut conformation to prevent specific and nonspecific self-associations, and is achieved by strict adherence to acidic to neutral pH during purification (Gill, 2012 ▶). Protein solutions are then stable as intact dimers for a week at room temperature or 277 K and no longer have to be stored by unquantifiable ammonium-sulfate precipitations. For longer-term storage, although a number of additives have been screened, cooling still induces a high polydispersity as judged by dynamic light-scattering measurements. Fresh protein must be purified for crystallization trials beyond these time limits. Moreover, even with a few crystals in hand, most cryoprotectants (traditional oils, PEGs, salts, alcohols) used in low-temperature X-ray data collection induced highly mosaic lattices, a problem fortuitously overcome by brief exposure to commercial bicycle oil, rapid cooling and annealing procedures as outlined here.

## 4. Conclusions

We have determined the space group of Δ1–62NtNBCe1-A crystals in order to implement viable refinement procedures on Nt crystals containing disease-causing mutations. Calculating twin fractions over small incremental resolution bins was useful to identify that the values arose from actual twinning, unlike those seemingly from full-length Nt crystals. Excluding the highest resolution shells, which appear to give artificially low values for the twin fraction, possibly owing to the larger measurement errors in this range, the trend demonstrates that the percentage twin fraction has a value in the low 30s after annealing and in the low 40s before annealing, assuming that the change in behavior can be attributed to the crystal treatment and not to variations in the crystal lattice. With structure determination of the Δ1–62NtNBCe1-A now possible, the role of the Nt in maintaining the interstitial pH and the effect of N-terminal truncation mutations can be detailed. From our earlier biophysical studies and low-resolution structure of full-length Nt, we predict that truncations at the extreme Nt eliminate part of a gate regulating access into a substrate tunnel that traverses the Nt and enters into the transmembrane domain, also causing loss of pH sensitivity, dimer–dimer self-associations or patch formation in the membrane, regulation of bicarbonate uptake into the Nt and possibly affecting other protein–protein interactions such as those needed for efficient trafficking to the membrane.

## Figures and Tables

**Table 1:** Δ1–62NtNBCe1 crystal parameters Values in parentheses are for the outermost resolution shell.

Radiation wavelength (Å)	1.1
Resolution range (Å)	34.1–2.41
Space group	*P*3_1_
Unit-cell parameters (Å, °)	*a* = *b* = 54.0, *c* = 398.4, β = 120
Mosaicity (°)	1.1
Completeness (%)	89.2 (70.8)
Observations	83208
Unique reflections	45122
Multiplicity	1.8 (1.4)
*R*_merge_ † (%)	6.3 (56.7)
〈*I*/σ(*I*)〉	18.6 (0.9)
Twin fraction (%)	33

† *R*_merge_ = 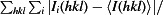 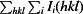, where *I_i_*(*hkl*) is the *i*th measurement and 〈*I*(*hkl*)〉 is the mean of all measurements of the intensity of the reflection with Miller indices *hkl*.

**Table 2:** Crystal packing

Space group	*Z*-score†
*P*3	No solution
*P*3_1_	15
*P*3_2_	3.0
*P*321	No solution
*P*3_1_21	7.6
*P*3_2_21	7.6
*P*312	No solution
*P*3_1_12	4.8
*P*3_2_12	4.8

† Calculated using the partial full-length Nt molecular probe.
